# Real-Time Use of a Dynamic Model To Measure the Impact of Public Health Interventions on Measles Outbreak Size and Duration — Chicago, Illinois, 2024

**DOI:** 10.15585/mmwr.mm7319a2

**Published:** 2024-05-16

**Authors:** Nina B. Masters, Inga Holmdahl, Paige B. Miller, Chirag K. Kumar, Catherine M. Herzog, Peter M. DeJonge, Stephanie Gretsch, Sara E. Oliver, Manisha Patel, David E. Sugerman, Beau B. Bruce, Brian F. Borah, Scott W. Olesen

**Affiliations:** ^1^Division of Viral Diseases, National Center for Immunization and Respiratory Diseases, CDC; ^2^Predict Division, Center for Forecasting and Outbreak Analytics, CDC; ^3^U.S. Digital Corps, Technology Transformation Services, General Services Administration, Washington, DC; ^4^Chicago Department of Public Health, Chicago, Illinois; ^5^Career Epidemiology Field Officer Program, CDC.

**Summary**

**What is already known about this topic?**

Measles is a highly infectious, vaccine-preventable disease. Fifty-seven measles cases were associated with residence in or contact with persons in a migrant shelter in Chicago, Illinois.

**What is added by this report?**

CDC developed dynamic models of shelter residents in real time to produce forecasts and assess the impact of interventions on outbreak size and duration. These models aided expectation-setting and resource planning and underscored the need for vaccination campaigns.

**What are the implications for public health practice?**

Real-time modeling can support public health response, set expectations about outbreak size and duration, and quantify the impact of interventions. Prompt mass vaccination and active case-finding likely substantially reduced the likelihood of a large measles outbreak in Chicago.

## Abstract

Measles is a highly infectious, vaccine-preventable disease that can cause severe illness, hospitalization, and death. A measles outbreak associated with a migrant shelter in Chicago occurred during February–April 2024, in which a total of 57 confirmed cases were identified, including 52 among shelter residents, three among staff members, and two among community members with a known link to the shelter. CDC simulated a measles outbreak among shelter residents using a dynamic disease model, updated in real time as additional cases were identified, to produce outbreak forecasts and assess the impact of public health interventions. As of April 8, the model forecasted a median final outbreak size of 58 cases (IQR = 56–60 cases); model fit and prediction range improved as more case data became available. Counterfactual analysis of different intervention scenarios demonstrated the importance of early deployment of public health interventions in Chicago, with a 69% chance of an outbreak of 100 or more cases had there been no mass vaccination or active case-finding compared with only a 1% chance when those interventions were deployed. This analysis highlights the value of using real-time, dynamic models to aid public health response, set expectations about outbreak size and duration, and quantify the impact of interventions. The model shows that prompt mass vaccination and active case-finding likely substantially reduced the chance of a large (100 or more cases) outbreak in Chicago.

## Introduction

Measles is an extremely infectious, vaccine-preventable febrile rash illness that can cause severe complications, including pneumonia, encephalitis, and death (*1*). Measles has a secondary attack rate among susceptible close contacts of >90%, making it one of the most infectious known diseases; prompt recognition and investigation of measles is important to limit its spread (*1*). On March 4, 2024, the Chicago Department of Public Health (CDPH) was notified of a suspected measles case in a resident of a temporary shelter for migrants primarily housing new arrivals from Venezuela. The shelter was a congregate setting with shared sleeping areas, with some rooms housing 500 or more residents (*2*). The patient had been hospitalized with suspected measles on February 27, after developing a rash on February 26, and was infectious in the shelter during February 22–27 (*2*). As of May 13, a total of 57 confirmed measles cases were identified, including 52 among shelter residents (with dates of rash onset ranging from February 26 through April 4, 2024), three among staff members, and two among community members with a known link to the shelter. Upon request from CDPH, CDC created dynamic measles models to forecast outbreak size and duration among shelter residents and quantitatively assess the impact of public health interventions.

## Methods

### Population Characteristics

CDC obtained measles outbreak data from CDPH multiple times per week. The population of the shelter as of March 8, the day interventions began, was 1,877 persons. Active case-finding was implemented on March 8, and during March 8–10, a total of 882 measles, mumps, and rubella (MMR) vaccine doses were administered to persons without proof of vaccination, bringing the final measles vaccine coverage among shelter residents to 93% (*2*). Rash onset date was missing for two patients; for these persons, symptom onset date was used to parameterize the model.

### Model Design

CDC adapted a model of measles transmission in a congregate setting previously developed during the 2021 Operation Allies Welcome (OAW) response (*3*). In this model, persons are placed into compartments representing their state relative to measles infection (i.e., susceptible, exposed, infected, and removed), age, and pregnancy status. Compared with the OAW model (*3*), this model also included a time-varying intervention representing active case-finding and a case ascertainment delay (Supplementary Figure, https://stacks.cdc.gov/view/cdc/155330). Model structure and parameterization evolved over the course of the outbreak; in this report, the retrospective predictions from the final model version are presented.*

### Statistical Methods

An age-specific measles immunity profile of Venezuela in 2024 was constructed using a previously described approach (*4*) (Supplementary Table 1, https://stacks.cdc.gov/view/cdc/155328). The effectiveness of a single MMR vaccine dose was assumed to be 84% for persons aged 6–11 months and 92.5% for persons aged ≥12 months (*5*). Natural history parameters for measles were derived from the literature and calibrated to available data using approximate Bayesian computation (Supplementary Table 2, https://stacks.cdc.gov/view/cdc/155329) (*6*). The model was simulated for 365 days beginning February 1, 2024, with 10,000 stochastic simulations per scenario.

Within each simulation, the model identified incident cases over time. Outbreak size forecasts were calibrated by selecting 100 simulations with the smallest absolute difference between predicted and observed daily cumulative measles cases among shelter residents. The model was run multiple times each week and fit to new data as outbreak cases were reported.

Counterfactual scenarios were simulated to determine potential outcomes if mass vaccination had started a week earlier (March 1, 2024) or a week later (March 15, 2024), as well as if active case-finding had not been implemented. Counterfactual outbreak trajectories were not calibrated to observed data. Simulations were conducted in R (version 4.3.0; R Foundation) using the adaptivetau package (*7*). This activity was reviewed by CDC, deemed not research, and was conducted consistent with applicable federal law and CDC policy.^†^

## Results

### Parameter Estimation for Public Health Response

The observed outbreak case series among shelter residents was found to be consistent with simulations using a basic reproduction number (R_0_, the average number of secondary cases that would result from a single case in a completely susceptible population) for measles of 25. The case series was also consistent with active case-finding leading to a 25% reduction in the infectious period of measles cases, from an average of 5 days to 3.8 days.

### Real-Time Dynamic Model Results During an Outbreak

Model fit and prediction range improved as more case data became available; uncertainty was higher, and accuracy was lower earlier in the outbreak (Table 1). Using data available as of March 18, 2024, with 18 cases reported among shelter residents, the model forecasted a median final outbreak size of 38 cases with a median final rash onset on April 18. Using data available as of March 25, with 47 cases reported among shelter residents, the model forecasted a median final outbreak size of 60 cases with a median final rash onset on April 20. On April 8, with 52 cases reported among shelter residents, the model forecasted a median final outbreak size of 58 cases.

**TABLE 1:** Predictive model iterations week over week among shelter residents based on available case data during measles outbreak associated with a migrant shelter — Chicago, Illinois, 2024

Date	No. of observed measles cases among shelter residents	Median model-predicted final outbreak size (IQR)	Median model-predicted final rash onset date	Relative % difference between median predicted and observed final outbreak size*
Mar 11	7	29 (20–39)	Apr 16	−44
Mar 18	18	38 (31–41)	Apr 18	−28
Mar 25	47	60 (57–65)	Apr 20	15
Apr 1	51	60 (58–63)	Apr 20	15
Apr 8	52	58 (56–60)	Apr 18	12

* Calculated by taking the percent difference between the predicted outbreak size as of each date and the final observed outbreak size among shelter residents (52). Negative numbers reflect underestimates of total outbreak size, and positive numbers reflect overestimates of total outbreak size.

### Counterfactual Analysis of Interventions

Counterfactual analyses of varied mass vaccination start dates and implementation or nonimplementation of active case-finding were performed (Table 2). If there had been no mass vaccination or active case-finding after the index case, the model predicted a 69% chance of an outbreak of 100 or more cases with an estimated median last rash onset occurring on May 26, 2024, which is consistent with a median outbreak size of 235 cases (IQR = 232–238) (Figure). In contrast, modeling CDPH’s interventions (i.e., a mass vaccination campaign and active case-finding beginning March 8), the model predicted a 1% chance of an outbreak of 100 or more cases with an estimated median last rash onset occurring on April 9, 2024, a sixty-nine-fold decrease compared with the scenario with no intervention. A 1-week delay in mass vaccination increased the chance of an outbreak of 100 or more cases to 8% with active case-finding (an eightfold increase compared with the interventions CDPH deployed) and 15% without. If case notification to public health had occurred earlier and the mass vaccination campaign had been initiated on March 1, the chance of an outbreak of 100 or more cases would have been zero, and the chance of an outbreak of 50–99 cases would have been 2% with active case-finding and 3% without.

**TABLE 2:** Counterfactual analysis of the impact of mass vaccination and active case-finding interventions on the chance of a large measles outbreak and median duration of a measles outbreak among shelter residents — Chicago, Illinois, 2024

Intervention start dates		Chance of additional measles cases among shelter residents,* %					Duration
Mass vaccination	Active case-finding	Zero cases	1–9 cases	10–49 cases	50–99 cases	≥100 cases	Last rash onset in outbreak (median)
Never	Never	24	7	—	—	69	May 26
Mar 15	Never	24	10	23	28	15	Apr 21
	Mar 8	23	13	28	27	8	Apr 17
Mar 8	Never	24	15	40	21	1	Apr 14
	Mar 8	23	17	43	17	1	Apr 9
Mar 1	Never	24	24	49	3	—	Apr 3
	Mar 8	23	26	49	2	—	Mar 30

* Number of measles cases beyond the index case.

**FIGURE:**
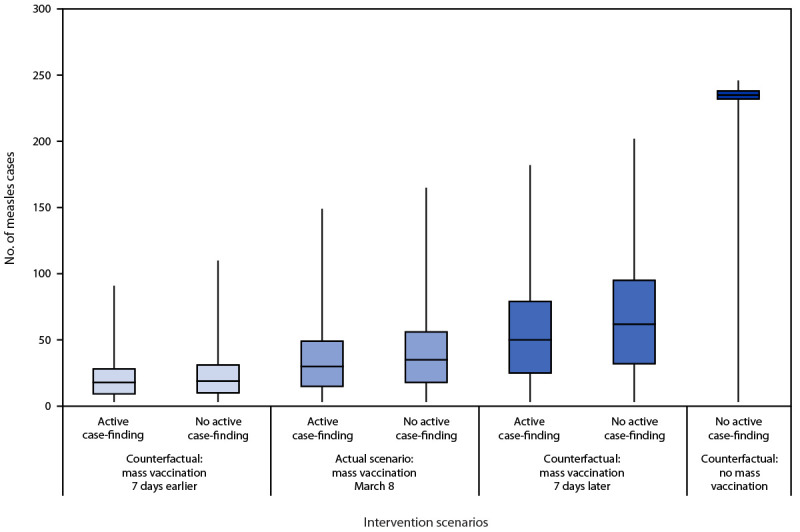
Counterfactual analysis* of the impact of mass vaccination and active case-finding interventions on the total number of measles cases among shelter residents, conditional on a measles outbreak^†^ associated with a migrant shelter — Chicago, Illinois, 2024 * Boxplots represent median (horizontal line) and 25th (bottom) and 75th (top) percentiles across stochastic simulations, and the error bars extend from the minimum to maximum values. ^†^ Three or more cases.

## Discussion

Dynamic disease models can be used and updated in real time to assist with public health response and resource planning as outbreaks unfold. During this measles outbreak, the model estimated high values of R_0_ (25), higher than the traditionally reported 12–18 (*8*), underscoring the potential for rapid transmission of measles and a high force of infection in a dense congregate setting. This parameter, estimated early during the outbreak and shared with partners in Chicago, emphasized the importance of active case surveillance because of the possibility of many secondary cases. CDPH used these findings, along with observed measles cases among shelter residents who had received a single dose of MMR vaccine, to demonstrate the need for continued vaccination campaigns during the outbreak, including a second dose campaign 28 days after the first dose, (*2*) and to recommend that shelter staff members and essential visitors provide evidence of immunity to measles.

The model provided an expectation of total outbreak size and duration weeks before the last rash onset occurred. CDPH shared model results with city leadership, other state and local government agencies, and health care partners to facilitate expectation-setting and resource planning. The model also highlighted the impact of early intervention on measles outbreaks in congregate settings, showing a sixty-nine-fold reduction (from 69% to 1%) in the chance of an outbreak of 100 or more cases and a median reduction in outbreak duration by 7 weeks when mass vaccination and active case-finding were initiated on March 8 compared with no intervention. If mass vaccination had been delayed by 1 week, there would have been an eightfold increase (from 1% to 8%) in the chance of an outbreak of 100 or more cases over the scenario in which mass vaccinations were deployed on March 8. These results are consistent with those from the OAW response, in which a modeled 7-day delay in vaccination would have yielded a 50% increase in median outbreak size (*3*). In addition, if public health notification of the index case had occurred when measles was first clinically suspected, an opportunity to implement mass vaccination would have occurred sooner, further reducing the chance of a large outbreak. Together, these results highlight the value of prompt mass vaccination and active case-finding to reduce the size and duration of measles outbreaks. Amid an increase in the number of measles cases reported during 2024 (*9*), development of rapid analytic tools to aid public health outbreak responses is critical.

### Limitations

The findings in this report are subject to at least four limitations. First, parameters have substantial uncertainty, and reasonable values, guided by published literature and expert opinion, had to be selected because the outbreak was not large enough to estimate all parameters with high precision. Variability in outputs was due to stochastic variation rather than to parameter uncertainty. Second, the model structure overestimated the variability in the infectious period by assuming that it is exponentially distributed across infected persons. Third, this model did not account for the relocation of 22 family units to a quarantine hotel on March 11–12, instead modeling the whole population together, and thus might have overestimated final outbreak size. Finally, although a measles susceptibility profile of Venezuela was used to estimate population susceptibility, the actual measles immunity status of the population was unknown.

### Implications for Public Health Practice

This outbreak occurred among persons in a dense congregate setting with vaccination coverage below the 95% threshold recommended for prevention of measles spread (*10*). This use of dynamic models, updated in real time as the outbreak unfolded, aided public health response, setting expectations about likely outbreak trajectory and timing. The code for these models is publicly available for use by jurisdictional and academic partners at https://github.com/CDCgov/measles-model-chicago-2024. In addition, this modeling framework quantitatively assessed the impact of interventions using counterfactual scenarios to show that prompt mass vaccination and active case-finding likely substantially reduced the chance of a large outbreak in Chicago.

## Footnotes

* Code used to produce the analyses in this report is available at https://github.com/CDCgov/measles-model-chicago-2024.

† 45 C.F.R. part 46.102(l)(2), 21 C.F.R. part 56; 42 U.S.C. Sect. 241(d); 5 U.S.C. Sect. 552a; 44 U.S.C. Sect. 3501 et seq.

## References

[1] Gastañaduy PA, Redd SB, Clemmons NS, Measles [Chapter 7]. In: Manual for the surveillance of vaccine-preventable diseases. Atlanta, GA: US Department of Health and Human Services, CDC; 2023. https://www.cdc.gov/vaccines/pubs/surv-manual/chpt07-measles.html

[2] Gressick K, Nham A, Filardo TD, Measles outbreak associated with a migrant shelter—Chicago, Illinois, February–May 2024. MMWR Morb Mortal Wkly Rep 2024;73:424–9. https://www.cdc.gov/mmwr/volumes/73/wr/mm7319a1.htm?s_cid=mm7319a1_w 38753539 10.15585/mmwr.mm7319a1 PMC11115429

[3] Masters NB, Beck AS, Mathis AD, Measles virus transmission patterns and public health responses during Operation Allies Welcome: a descriptive epidemiological study. Lancet Public Health 2023;8:e618–28. 10.1016/S2468-2667(23)00130-5 37516478 PMC10411127

[4] World Health Organization. Meeting of the Advisory Committee on Immunization and Vaccines related Implementation Research (IVIR-AC). Geneva, Switzerland; World Health Organization; 2021. https://terrance.who.int/mediacentre/data/sage/210907-IVIR-AC-Pink-Book-August2021.pdf

[5] Uzicanin A, Zimmerman L. Field effectiveness of live attenuated measles-containing vaccines: a review of published literature. J Infect Dis 2011;204(Suppl 1):S133–49. 10.1093/infdis/jir102 21666154

[6] Cao Y, Gillespie DT, Petzold LR. Adaptive explicit-implicit tau-leaping method with automatic tau selection. J Chem Phys 2007;126:224101. 10.1063/1.2745299 17581038

[7] Sunnåker M, Busetto AG, Numminen E, Corander J, Foll M, Dessimoz C. Approximate Bayesian computation. PLOS Comput Biol 2013;9:e1002803. 10.1371/journal.pcbi.1002803 23341757 PMC3547661

[8] Guerra FM, Bolotin S, Lim G, The basic reproduction number (R_0_) of measles: a systematic review. Lancet Infect Dis 2017;17:e420–8. 10.1016/S1473-3099(17)30307-9 28757186

[9] Mathis AD, Raines K, Masters NB, Measles—United States, January 1, 2020–March 28, 2024. MMWR Morb Mortal Wkly Rep 2024;73:295–300. 10.15585/mmwr.mm7314a1 38602886 PMC11008791

[10] Orenstein WA, Hinman AR, Papania MJ. Evolution of measles elimination strategies in the United States. J Infect Dis 2004;189(Suppl 1):S17–22. 10.1086/377694 15106084

